# Prevalence of Obstructive Sleep Apnea Symptoms Among the Adult Population in Al-Ahsa, Saudi Arabia

**DOI:** 10.7759/cureus.31082

**Published:** 2022-11-04

**Authors:** Anwar Alsultan, Muthana Al Sahlawi, Mohammed Agha

**Affiliations:** 1 Internal Medicine, King Faisal University, Al-Ahsa, SAU; 2 Internal Medicine, King Faisal University, Al-Hasa, SAU; 3 Chest Disease and Tuberculosis, Menoufia University, Al Minufiyah, EGY

**Keywords:** al-ahsa, epworth sleepiness scale, prevalence, adult population, obstructive sleep apnea

## Abstract

Introduction

Obstructive sleep apnea (OSA) is a disorder characterized by repeated episodes of partial or complete obstruction of the airway during sleep. OSA can lead to serious long-term complications if left untreated.

Aim

This study aims to assess the prevalence of OSA symptoms among the adult population in Al-Ahsa, Saudi Arabia.

Subjects and methods

This is a cross-sectional study including the adult population living in Al-Ahsa, Saudi Arabia. A self-administered questionnaire was distributed to the targeted population using an online survey. The questionnaire was divided into two sections, where the first part was about the characteristics of the participant (i.e., age, gender, marital status, etc.), and the second part was the Epworth Sleepiness Scale (ESS) questionnaire to evaluate OSA symptoms.

Results

Three hundred and sixty adult subjects participated in this study by responding to the questionnaire (58.1% males vs. 41.9% females). The respondents' median age was 30 years old. The prevalence of OSA symptoms was 26.9%. In terms of OSA symptom severity, mild, moderate, and severe excessive daytime sleepiness were found among 12.5%, 8.3%, and 6.1%, respectively. The prevalence of OSA symptoms was significantly higher in the older group (>30 years; p=0.004), married participants (p=0.008), and obese or overweight (BMI ≥25 kg/m2; p=0.002). Multivariate regression estimates showed that being obese or overweight (BMI ≥25 kg/m2) was the sole independent significant predictor associated with increased odds of OSA symptoms.

Conclusion

The prevalence of OSA symptoms among Al-Ahsa residents was 26.9% with prevalence being higher in males than females. Further investigations are needed to establish the prevalence of OSA and understand its influence on the adult population in our region. People who were suspected to have OSA in this study should be reassessed using polysomnography to confirm the diagnosis. Patients with suspected OSA should be encouraged to adopt lifestyle modifications specifically targeted to/focused on weight reduction and smoking cessation.

## Introduction

Obstructive sleep apnea (OSA) is a disorder characterized by repeated episodes of partial or complete obstruction of the airway during sleep [1]. OSA is associated with cardiovascular diseases [2], hypertension [3], and diabetes mellitus [4]. OSA has also been recognized as a risk factor for motor vehicle crashes [5]. The prevalence of OSA in the middle-aged population was first estimated in 1993 by the ongoing population-based Wisconsin Sleep Cohort study, with a sample of 625 employed adults. The researchers found that 9% of women and 24% of men had at least five apneas or hypopneas per hour of sleep. When excessive daytime sleepiness was included as a criterion, the prevalence of OSAS was reported at 2% in females and 4% in males [6]. Nearly 80% of men and 93% of women with moderate to severe sleep apnea are believed to be undiagnosed [7].

The prevalence of obstructive sleep apnea symptoms in the middle‑aged population was measured between December 2005 and March 2006 at King Khalid University and the King Fahd National Guard Primary Health Clinics in Riyadh, Kingdom of Saudi Arabia. The study showed a high prevalence of symptoms of OSA [8]. In the primary care setting, one in three middle-aged Saudi males is at risk for OSA [8].

Level 1 polysomnography (PSG) is the gold standard test for the diagnosis of OSA. However, compared to the high estimated prevalence of OSA, the availability of sleep labs is much scarce. Therefore, the utilization of screening tools becomes necessary to classify patients based on their clinical symptoms, physical findings, risk factors, and comorbid illness into high‑ or low‑risk groups [9].

Among these tools is the Epworth Sleepiness scale (ESS). It is a simple, self-administered questionnaire that is shown to provide a measurement of the subject's general level of daytime sleepiness [10]. The consequences of OSA are either to the patient or to the community. Diagnosis of OSA is dependent on symptoms either from the patient or from his or her partner. The ESS is a simple and easy method to screen the presence of probability of OSA, especially in health sectors lacking polysomnography. We used this questionnaire as it is the widely used questionnaire in our region, especially with an approved Arabic copy of it. This study aims to assess the prevalence of OSA symptoms to put an alarm to our community about this underestimated disease and its consequences. Few published studies addressed OSA symptoms in Saudi Arabia, and no previous studies have estimated the prevalence of OSA symptoms in Al-Ahsa. The primary aim of this study is to assess the prevalence of obstructive sleep apnea symptoms among adult populations in Al-Ahsa.

## Materials and methods

This is a cross-sectional study including the adult population living in Al-Ahsa, Saudi Arabia. A sample size of 385 was calculated by keeping a confidence interval of 95% and a margin of error of 5%. Both males and females who are 18 or older and are residents of Al-Ahsa were included in the study. People younger than 18 and those who don't reside In Al-Ahsa were excluded. Data were collected using a self-administered questionnaire that was distributed to the targeted population using a Google Form (Google, Mountain View, California) in the Arabic language to increase the response rate. The questionnaire was divided into two sections, where the first part was about the characteristics of the participant (age, gender, marital status, nationality, education level, living area, weight, height, smoking habits, chronic diseases, medication usage), and the second part was the ESS questionnaire to assess the prevalence of OSA. The ESS is composed of eight items. The total knowledge score for all questions was 24. Each item has a four-point Likert scale ranging from 0 (would never doze) to three (high chance of dozing). ESS scores can be interpreted as follows: 0-5 lower normal daytime sleepiness, 6-10 higher normal daytime sleepiness, 11-12 mild excessive daytime sleepiness, 13-15 moderate excessive daytime sleepiness, and 16-24 severe excessive daytime sleepiness.

Statistical analysis

The median age (30 years) was used as a cutoff point when comparing the prevalence of OSA symptoms. Regarding BMI level, according to WHO recommendation, below 18.5 kg/m2 was considered as underweight, 18.5-24.9 kg/m2 was considered as normal weight, 25-29.9 kg/m2 was considered as overweight, and 30 kg/m2 or more was considered as obese. A cutoff point of 25 kg/m2 was used to categorize normal/underweight and obese/overweight.

Categorical variables were presented as numbers and percentages, while continuous variables were summarized as mean and standard deviation. The association between OSA with the sociodemographic characteristics of the participants was calculated using the Chi-squared test. Values returned as significant were then placed in a multivariate regression model to determine the independent significant factor associated with positive OSA symptoms. A p-value of 0.05 was considered statistically significant. The data were analyzed using Statistical Packages for Social Sciences (SPSS) version 26 (IBM Inc., Armonk, New York).

Ethical considerations

Ethical approval was taken from the Deanship of Scientific Research, Vice Presidency for Graduate Students and Scientific Research, King Faisal University. The participants were provided with study objectives, and informed consent was taken before participating in the study questionnaire. Participants were explained about study objectives, which were mentioned before the start of the questionnaire. They were free to accept or decline, and all were reassured that the information obtained would be kept anonymous and confidential.

## Results

We received 385 survey responses from the study population; 25 of them were excluded for not meeting the required criteria, giving an overall response rate of 93.5%. Table 1 presents the sociodemographic characteristics of the sample population living in Al-Ahsa. The most common age group was 18-25 years old (39.2%), with 58.1% being females and mostly being Saudis (98.3%). Married participants constitute 57.8%, and participants with bachelor’s degrees constitute 69.7%. Patients who were obese were 31.7% of the study population. The prevalence of smoking participants was 12.2%. The use of medications was reported by five respondents; two cases were using anxiety medications, such as benzodiazepines, and three cases were using sedatives like barbiturates.

**Table 1:** Participants’ sociodemographic characteristics (N=360)

Study variables	n (%)
Age group in years	
18-25 years	141 (39.2%)
26-35 years	75 (20.8%)
36-45 years	88 (24.4%)
>45 years	56 (15.6%)
Gender	
Male	209 (58.1%)
Female	151 (41.9%)
Nationality	
Saudi	354 (98.3%)
Non-Saudi	6 (01.7%)
Marital status	
Single	138 (38.3%)
Married	208 (57.8%)
Divorced	14 (03.9%)
Education level	
Primary school	3 (0.80%)
Middle school	15 (04.2%)
High school	76 (21.1%)
Bachelor	251 (69.7%)
Postgraduate	15 (04.2%)
BMI level	
Underweight (<18.5 kg/m2)	19 (05.3%)
Normal (18.5-24.9 kg/m2)	129 (35.8%)
Overweight (25-29.9 kg/m2)	98 (27.2%)
Obese (≥30 kg/m2)	114 (31.7%)
Smoking status	
Smoker	44 (12.2%)
Non-smoker	316 (87.8%)
Use of medications	
Anxiety medications (such as benzodiazepines)	2 (0.60%)
Sedative medications (such as barbiturates)	3 (0.80%)
None	355 (98.6%)

**Figure 1:**
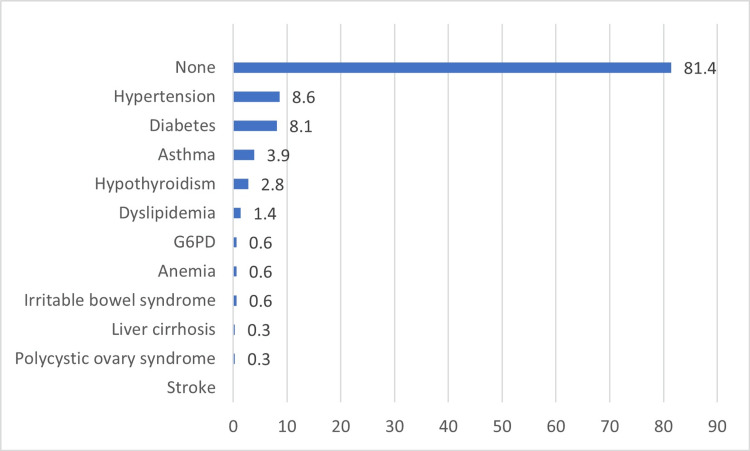
Associated chronic diseases

Figure 1 depicts associated chronic diseases. It can be observed that the most commonly known associated chronic disease was hypertension (8.6%), followed by diabetes (8.1%), and asthma (3.9%).

**Figure 2:**
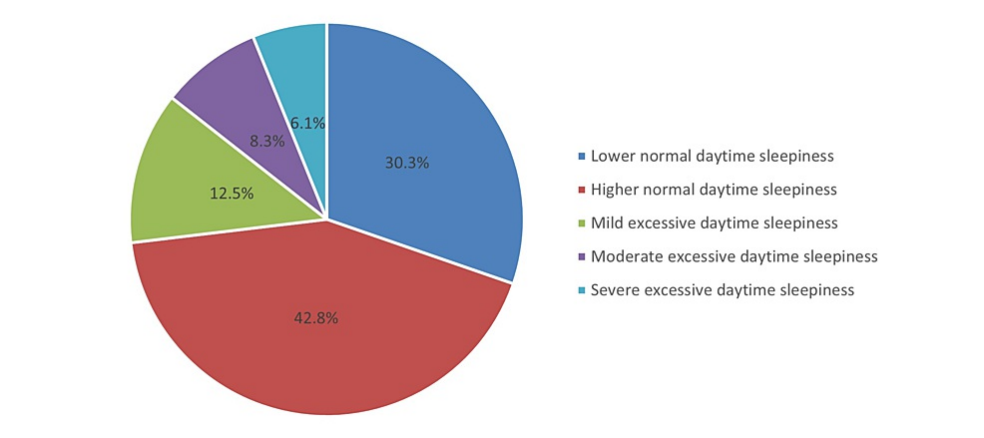
Severity of OSA as per ESS questionnaire criteria ESS - Epworth Sleepiness Scale, OSA - obstructive sleep apnea

Table 2 shows the prevalence of OSA. It was revealed that the overall mean score of ESS was 8.07 (SD 4.53). Based on the given criteria, positive OSA symptoms were found among 26.9%, and the rest were negative (73.1%). Regarding OSA severity, moderate excessive daytime sleepiness was detected among 8.3%, whereas severe excessive daytime sleepiness was detected in 6.1% of participants (see also Figure 2).

**Table 2:** Prevalence of obstructive sleep apnea according to the Epworth Sleepiness Scale (n=360) ESS - Epworth Sleepiness Scale, OSA - obstructive sleep apnea

ESS variables	n (%)
ESS score (mean ± SD)	8.07 ± 4.53
Symptoms of OSA	
Positive (score ≥11)	97 (26.9%)
Negative (score 0-10)	263 (73.1%)
Level of OSA	
Lower normal daytime sleepiness	109 (30.3%)
Higher normal daytime sleepiness	154 (42.8%)
Mild excessive daytime sleepiness	45 (12.5%)
Moderate excessive daytime sleepiness	30 (08.3%)
Severe excessive daytime sleepiness	22 (06.1%)

We used the Chi-squared test in Table 3 to measure the association between the symptoms of OSA and the sociodemographic characteristics of participants. Based on the results, it was observed that age group in years (X2=8.182; p=0.004), marital status (X2=6.943; p=0.008), and BMI level (X2=9.666; p=0.002) showed a positive association with the symptoms of OSA while gender, educational level, smoking status, and associated chronic disease did not show significant association with the symptoms of OSA (p>0.05).

**Table 3:** Association between the symptoms of OSA and the sociodemographic characteristics of participants (n=360) OSA - obstructive sleep apnea § p-value was calculated using the Chi-squared test,  ** Significant at p<0.05 level

Factor	Symptoms of OSA		X2	p-value ^§^
	Positive N (%), n=97	Negative N (%), n=263		
Age group in years				
≤30 years	37 (38.1%)	145 (55.1%)	8.182	0.004 **
>30 years	60 (61.9%)	118 (44.9%)		
Gender				
Male	60 (61.9%)	149 (56.7%)	0.787	0.375
Female	37 (38.1%)	114 (43.3%)		
Marital status				
Unmarried	30 (30.9%)	122 (46.4%)	6.943	0.008 **
Married	67 (69.1%)	141 (53.6%)		
Education level				
High school or below	21 (21.6%)	73 (27.8%)	1.37	0.242
Bachelor or higher	76 (78.4%)	190 (72.2%)		
BMI level				
Normal or underweight (<25 kg/m2)	27 (27.8%)	121 (46.0%)	9.666	0.002 **
Obese or overweight (≥25 kg/m2)	70 (72.2%)	142 (54.0%)		
Smoking status				
Smoker	15 (15.5%)	29 (11.0%)	1.301	0.254
Non-smoker	82 (84.5%)	234 (89.0%)		
Associated chronic disease				
Yes	21 (21.6%)	46 (17.5%)	0.809	0.368
No	76 (78.4%)	217 (82.5%)		

A multivariate regression analysis was subsequently performed to determine the independent significant predictor of positive OSA symptoms. We found that BMI level was the only significant predictor associated with OSA symptoms positive, which indicates that the risk of having OSA symptoms in participants who were obese or overweight (BMI ≥25 kg/m2) was predicted to be at least 1.8 times higher compared to participants who were normal or underweight (adjusted odds ratio (AOR)=1.846; 95% CI=1.078-3.159; p=0.025). On the other hand, although the odds of having OSA symptoms positive among the older age group (>30 years) were predicted to increase by at least 1.3 times higher (AOR=1.337; 95% CI=0.718 - 2.488; p=0.360) and among married participants by at least 1.4 times higher (AOR=1.405; 95% CI=0.752 - 2.626; p=0.286), however, the overall results did not reach statistical significance (Table 4).

**Table 4:** Multivariate regression analysis to determine the independent significant factor associated with positive OSA symptoms (n=360) AOR - adjusted odds ratio, CI - confidence interval, OSA - obstructive sleep apnea ** Significant at p<0.05 level

Factor	AOR	95% CI	p-value
Age group in years			
≤30 years	Ref		
>30 years	1.337	0.718-2.488	0.36
Marital status			
Unmarried	Ref		
Married	1.405	0.752-2.626	0.286
BMI level			
Normal or underweight (<25 kg/m2)	Ref		
Obese or overweight (≥25 kg/m2)	1.846	1.078-3.159	0.025 **

## Discussion

The present study investigated the prevalence of OSA symptoms among the Al-Ahsa population and potential risk factors. The prevalence of OSA symptoms using the ESS questionnaire was high. 26.9% out of 360 Al-Ahsa residents appeared to have OSA symptoms, with prevalence being higher in men (61%) than in women (38.1%). Consistent with our findings, in a study conducted in Riyadh [11] using the Berlin Questionnaire (BQ), the prevalence of OSA symptoms was 21.7%, wherein the prevalence was higher in males (78.3%) than in females (21%). However, in Jeddah, Saudi Arabia, using a similar questionnaire, [12] the author discovers that Saudi pilots had a greater prevalence of OSA, with prevalence rates of 69% according to home sleep testing, majority of which had mild OSA (64%), and 5% had moderate and severe OSA (2.5% each).

Moreover, in a systematic review published in Kyrgyzstan [13], where the authors evaluated the prevalence of OSA in Asian adults diagnosed via sleep monitoring and the prevalence of patients at risk for OSA as assessed by symptomatology and/or sleep questionnaires, the OSA prevalence rate among the Asian population has a range from 3% to 97.3%. However, due to the lack of data on some Asian countries, their findings were not deemed conclusive. Contrary to these reports, in another systematic review done in Australia [14], the prevalence range was lower than that of Asia. The variations in the prevalence of OSA among the general population could be due to the different methods of diagnosing the disorder. But in a study conducted by Dixit et al., the effectiveness of BQ and ESS questionnaires was compared among adult patients with signs, symptoms, and history of OSA. According to their outcome, BQ is a more valid, reliable, and sensitive parameter to screen patients for OSA and may help in improving the quality of life of such patients, whereas ESS is a less sensitive diagnostic tool [9]. Notwithstanding previous reports, Franklin and Linberg [15] conferred that the deviations in the prevalence of OSA are maybe due to the different diagnostic criteria, definitions, study methodology, and characteristics of included populations.

Mild excessive daytime sleepiness was detected among 12.5% of our population, 8.3% was moderate, and the rest (6.1%) was severe. Like the study in Jeddah, Saudi Arabia, excessive daytime sleepiness was reported by 23.1% of the Saudi pilots [12]. The author explained that Saudi airline pilots and first officers are prone to this kind of disorder due to the airline crews are required to cope with very early and very late flights, traversing different time zones, and sleep deprivation leading to fatigue and increasing physical and mental health disorders. This is consistent with this study, which found that 14.6% of pilots were obese and that overweight was prevalent among them at 53.7%. Pilots who had worked nights for six to ten years and had a hard time unwinding after work, when perceived morningness was a protective factor, were most likely to be overweight. Working nights for six to ten years, having trouble relaxing after work, sleeping for less than six hours on days off, having other disorders that have been recognized, and engaging in less than 150 minutes of physical activity each week were all risk factors for obesity. [16] In our univariate analysis, age was a factor in OSA symptoms, where the older age group (>30 years) had a higher OSA prevalence than the younger age group (≤30 years). This is consistent with the report of Wali et al. [17]. According to their reports, age of 50 years or higher was one of the predictors of OSA symptoms; other reported independent risk factors of OSA were gender male, obesity, and history of hypertension.

The association between OSA and age had also been accounted for by Khan et al. [18]. However, they found no significant relations between OSA and gender, which was consistent with our reports. Similarly, we noted that marital status was also a relevant factor in OSA, with married participants being seen to develop the disorder more than unmarried participants, which concurred with the report of Sogebi and Ogunwale [19]. This is consistent with the report of Jeler [20]. Patients who have a stable life partner are more likely to have OSA identified, with a relatively high percentage of married patients (81.2%) having the condition compared to patients who did not have a stable life partner (unmarried, divorced, widowed). This is due to the patient’s attention to nocturnal symptoms, such as snoring, apnea episodes, and insomnia.

Several papers indicated obesity as one of the major risk factors for OSA [11,13-15,19]. This had also been detected in our study. Based on our regression estimates, overweight and obese participants are predicted to increase the risk of developing OSA by at least 1.8 times compared to participants with normal and underweight BMI. In Riyadh, Saudi Arabia [11], the odds of OSA were even higher than our result, wherein obese patients were 10-fold higher to report OSA compared to non-obese patients. The excess fat tissue around the neck might cause airway narrowing which is related to obesity. According to a report [21], Australian adults reported severity of sleep-disordered breathing increased rapidly in two decades, with an increase annually in median BMI of 0.15 kg/m2 for males and 0.14 kg/m2 for females, which supports a theory that worsening sleep-disordered breathing is likely associated with elevated BMI, primarily obesity. This indicates the need for lifestyle modifications among obese patients who are at higher risk of developing OSA, such as weight loss programs and dietary methods to address the increasing trend of OSA, leading to an improved quality of life among this group of patients.

## Conclusions

The prevalence of OSA among Al-Ahsa residents was 26.9%, with prevalence being higher in males than females. Elevated BMI was determined as an independent risk factor for OSA symptoms. Preventive strategies to address the high prevalence of OSA symptoms are necessary to decrease the prevalence of this disorder in our population. Healthcare professionals should devise a more clinical approach to diagnosing patients who are at high risk for OSA and provide necessary interventions such as lifestyle modifications which could serve as compelling means of eliminating the disorder. Further investigations are needed in order to establish the prevalence of OSA and understand its influence on the adult population in our region. People who are suspected to have OSA symptoms in this work should be reassessed using polysomnography to confirm the diagnosis. Patients with suspected OSA should be encouraged to weight reductio and smoking cessation, and lifestyle modification should be planned.
